# Effects of replacing 50% of grass-clover silage with whole-crop wheat silage on feed intake, growth, and enteric methane emissions in dairy heifers

**DOI:** 10.3168/jdsc.2026-1098

**Published:** 2026-11-03

**Authors:** Markos Managos, Cecilia Lindahl, Sigrid Agenäs, Mikaela Lindberg

**Affiliations:** 1 Department of Applied Animal Science and Welfare, Swedish University of Agricultural Sciences, 750 07 Uppsala, Sweden; 2 Lantmännen Lantbruk, 205 03 Malmö, Sweden

## Abstract

Replacement heifers contribute substantially to greenhouse gas emissions from dairy systems, yet information on emissions from heifers fed diets containing whole-crop cereal silage as an alternative forage remains limited. This study evaluated feed intake, growth, and enteric methane emissions in Swedish Holstein and Nordic Red heifers fed either grass-clover silage or a 50:50 mix of grass-clover and whole-crop wheat silage. Twenty-two pregnant heifers (mean age ± SD; 18.3 ± 1.16 mo) were randomly assigned to treatments in a randomized complete block design. Feed intake and methane emissions (GreenFeed system, C-Lock Inc.) were monitored throughout the covariate (2 wk), adaptation (1 wk), and experimental (4 wk) periods, whereas body weight and backfat thickness were measured at the start and end of the study. Dry matter intake, methane emissions, and average daily gain or backfat thickness did not differ between diets. These findings suggest that whole-crop wheat silage can partially replace grass-clover silage in the diets of pregnant dairy heifers without affecting feed intake, growth rate, or enteric methane emissions.

## Graphical Abstract

**Figure:**
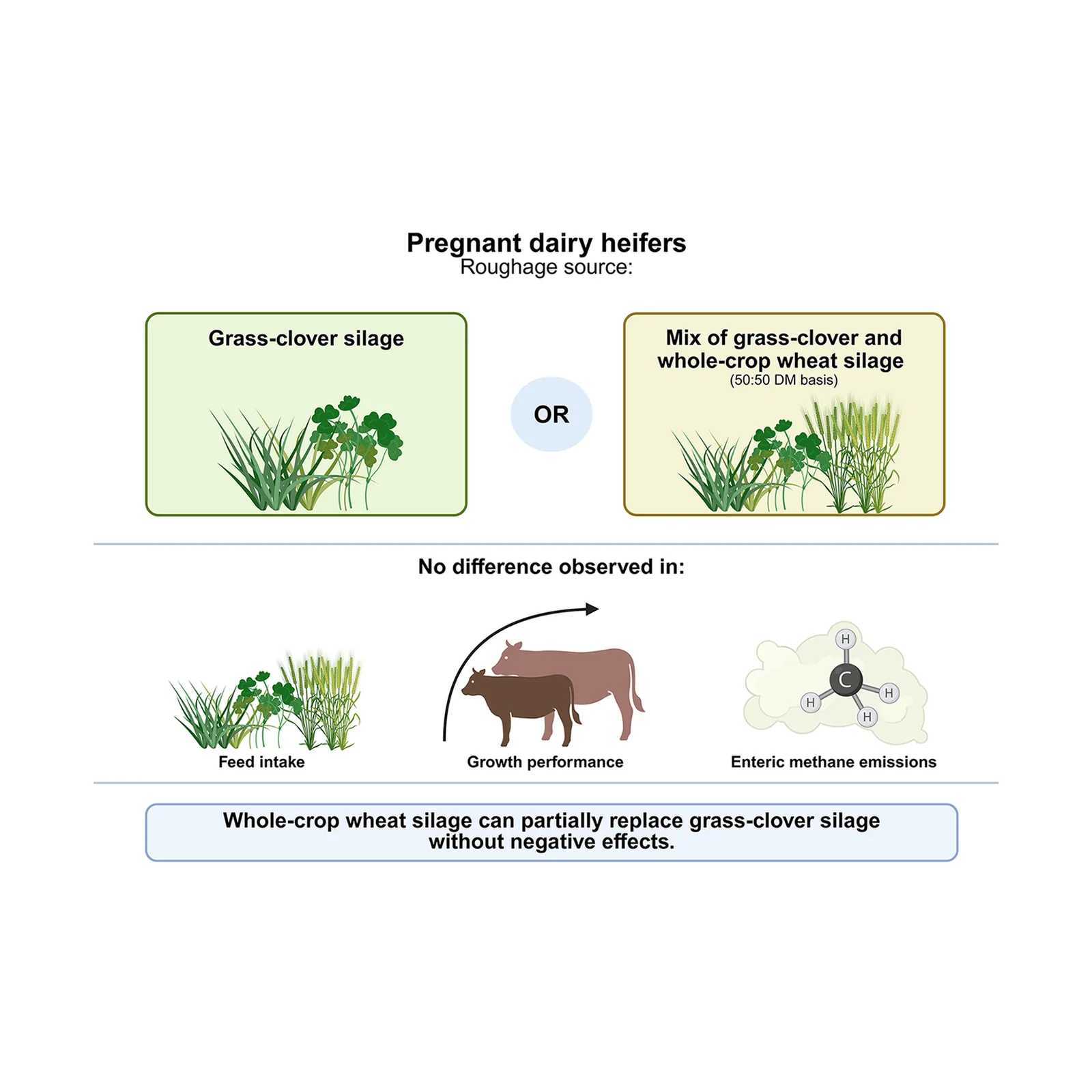

Adequate roughage sources are essential for the successful rearing of replacement heifers and for supporting their growth and development. Swedish dairy herds have an annual replacement rate of ∼36% (Växa Sverige, 2025), making heifer rearing an important contributor to feed costs, dairy herd productivity, profitability, and GHG emissions (Henriksson, 2014; Clasen et al., 2024; Sommerseth et al., 2024). Common crop rotations include ley and cereals, with grass-clover silage (**GS**) as the primary roughage source for dairy farming (Swedish Board of Agriculture, 2023). However, extreme weather events, such as the 2018 summer drought in Sweden, can severely limit feed availability, affecting roughage supplies. In 2018, farmers responded by harvesting cereals earlier and conserving them as whole-crop silage, prioritizing GS for high-producing dairy cows while feeding ensiled whole-crop cereals to replacement heifers and low-producing cows (Statistics Sweden, 2018; Spörndly et al., 2019).

Although whole-crop cereal silage has been nutritionally evaluated in dairy cattle diets, demonstrating effects on feed intake, nutrient digestibility, and growth performance (Wallsten et al., 2009, 2010; Rustas et al., 2011), its effects on enteric CH_4_ emissions remain unclear. Whole-crop wheat silage differs from GS in both fiber and starch concentrations, which may exert opposing effects on rumen fermentation and enteric CH_4_ production. This creates a knowledge gap, as existing CH_4_ prediction models and life-cycle assessments (**LCA**) are primarily based on experiments using grass or maize silage diets. Therefore, this study investigated the effects of partially replacing GS with whole-crop wheat silage on enteric CH_4_ emissions, feed intake, and growth performance in dairy replacement heifers. We hypothesized that replacing part of the GS with whole-crop wheat silage would alter enteric CH_4_ emissions while having only limited effects on feed intake and growth performance.

The experiment was conducted at Lantmännen's experimental dairy farm “Nötcenter Viken” in Falköping, Sweden, from August to October 2024. A randomized complete block design was used. Twenty-two pregnant dairy heifers (14 Swedish Holstein and 8 Nordic Red) were divided into 11 breed- and BW-matched blocks and randomly assigned to 1 of 2 dietary treatments (mean ± SD; age: 18.3 ± 1.16 mo; gestation: 3.9 ± 0.95 mo). The study included a 2-wk covariate period, a 1-wk dietary adaptation period, and 4 experimental weeks. All heifers were housed in a freestall barn with 22 cubicles, rubber-mat flooring, peat bedding, and shared access to a single GreenFeed unit (C-Lock Inc., Rapid City, SD).

Feed and water were provided ad libitum (10% refusals), with individual daily feed intake recorded automatically using scales equipped with feed mangers (BioControl, CRFI, Rakkestad, Norway). The dietary treatments included (1) GS, consisting of a third cut of grass-clover ley, and (2) a roughage mix (**RM**), consisting of equal parts (DM basis) of grass and whole-crop wheat silage. Both treatments were supplemented with 8 g of mineral mix (Effekt Fruktsam, Lantmännen, Malmö, Sweden) and 4 g NaCl per kg DM. The diets were mixed daily, using a stationary mixer (Feed Mixer-Multimix, Cormall, Sønderborg, Denmark) and offered ad libitum once daily via an automatic feeder (Free Stall Feeder M2000 XL, GEA, Düsseldorf, Germany). Silage DM content was assessed twice weekly to adjust RM composition, and dried sugar beet pulp pellets (Betfor, Nordic Sugar AB, Malmö, Sweden) were provided through the GreenFeed system. The chemical composition of the feeds is detailed in Table 1.

**Table 1:** Chemical composition of feed ingredients and estimated dietary forage mixtures (mean ± SD; g/kg DM unless otherwise stated; n = 4 samples per feed ingredient)^1^

Parameter	Betfor	Silage sample		Dietary forage mixture^2^	
		Grass-clover	Whole-crop wheat	GS	RM
DM (g/kg fresh matter)	926 ± 2.7	335 ± 27.5	425 ± 38.4	338 ± 27.5	383 ± 27.1
Ash	67 ± 4.0	104 ± 5.7	51 ± 0.8	115 ± 5.7	87 ± 1.7
AIA	19 ± 3.2	17 ± 1.8	13 ± 6.5	17 ± 1.7	15 ± 2.0
CP	80 ± 1.7	184 ± 4.7	103 ± 5.4	182 ± 4.8	141 ± 4.5
aNDFom	335 ± 3.8	499 ± 16.6	553 ± 9.9	493 ± 16.6	511 ± 10.7
Starch	9.5 ± 2.59	3.0 ± 3.82	123 ± 10.3	3.0 ± 3.82	77 ± 10.6
pH		4.4 ± 0.11	4.8 ± 0.28		

1 AIA = acid-insoluble ash; aNDFom = amylase-treated NDF, expressed exclusive of residual ash; GS = grass-clover silage roughage mix; RM = 50:50 DM basis mixture of GS and whole-crop wheat silage; Betfor = dried molassed sugar beet pulp pellets (Nordic Sugar AB, Malmö, Sweden).

2 Estimated chemical composition of GS and RM forage mixtures (50:50 DM basis of grass-clover and whole-crop wheat silage), including 8 g mineral mix and 4 g NaCl per kg DM.

During experimental wk 1 and 4, forage and concentrate were sampled Monday through Thursday. In wk 2 and 3, silage was sampled Monday through Friday, and concentrates were sampled on Monday and Thursday. All samples were stored at −20°C and pooled by dietary treatment and week. Feed samples were analyzed for DM, ash, amylase-treated NDF exclusive of residual ash (**aNDFom**), CP, starch, pH, and acid-insoluble ash (**AIA**), whereas fecal subsamples were freeze-dried, milled, and analyzed for DM, ash, aNDFom, CP, and AIA according to the methods reported by Managos et al. (2025), with AIA determination according to Van Keulen and Young (1977). Fecal grab samples were collected rectally once daily for 3 d during wk 1 and wk 4 (Mehtiö et al., 2016). Apparent total-tract digestibility of DM, OM, aNDFom, and CP was estimated using AIA as an internal marker.

Body weight was recorded on 2 consecutive days, and backfat thickness (**BFT**) once during the covariate period and at the end of the experiment. Five repeated measurements per individual and sampling occasion were collected using a LOGIQ e Ultrasound (GE HealthCare) with a 12L-RS wide-band linear array transducer, positioned perpendicular to the skin at one-third of the distance from the tuber ischii to the tuber coxae on the left side (Schröder and Staufenbiel, 2006). Exhaled CO_2_ and CH_4_ emissions were measured throughout the whole experiment. Heifers voluntarily accessed the GreenFeed with a minimum 5-h interval between visits and were attracted using pelleted sugar beet pulp (maximum of 8 drops per visit; 1 drop every 40 s; 39.1 ± 3.18 g per drop). The system was automatically calibrated twice weekly using manufacturer-supplied standard gases, and CO_2_ recovery tests were conducted every third week, yielding a mean recovery of 100.4%.

Data were analyzed in RStudio (R Core Team, 2022; Posit Team, 2024) using the tidyverse package (Wickham et al., 2019). Data from GreenFeed were filtered to include only successful visits (duration ≥3 min; average airflow >35 L/s) and to exclude animals with fewer than 20 successful visits during the experimental period (Manafiazar et al., 2016; Arthur et al., 2017). Consequently, gas emission analyses included 17 heifers (GS: 8; RM: 9) with an average (±SD) of 80 ± 11.2 and 67 ± 21.1 successful visits per heifer during the experimental period for the GS and RM treatments, respectively (Table 2). Weekly BW was estimated by assuming a linear change between the BW measurements collected at the start and end of the experiment, and ADG was calculated accordingly. Intake, digestibility, BW, and ADG analyses included all 22 heifers (11 per treatment), whereas 1 RM heifer had missing BFT measurements. Gas emission and feed intake data were averaged by heifer and week before statistical analysis. Data were analyzed using linear mixed-effects models with treatment, week, their interaction, and block as fixed effects and animal as a random effect, using the “lme” function of the “nlme” package (Pinheiro et al., 2022). Initial BW was included as a covariate for BW, BFT, DMI/BW, CH_4_/BW, and CO_2_/BW, whereas DMI, forage intake, and Betfor intake were included as covariates for CH_4_, CO_2_, CH_4_/DMI, and CO_2_/DMI, where appropriate. Repeated measures were modeled using an autoregressive covariance structure. ANOVA was performed using the car package with type III sums of squares and the Kenward-Roger degrees-of-freedom adjustment (Fox and Weisberg, 2019). Treatment × week interactions were removed from the model if nonsignificant via backward stepwise selection, based on likelihood ratio tests, Akaike information criterion, and Bayesian information criterion. Residuals were assessed for normality, and transformations were applied when necessary. Statistical significance was set at *P* < 0.05, and pairwise comparisons were adjusted using Tukey's method and the “emmeans” package (Lenth, 2023).

**Table 2:** Effects of grass-clover silage (GS) and a roughage mix (RM; 50:50 DM basis of whole-crop wheat silage and GS) on feed intake, nutrient digestibility, growth performance, and enteric gas emissions of pregnant Holstein and Nordic Red heifers (estimated marginal means, SEM, and *P*-values)^1^

Parameter	Diet		SEM^2^	*P*-value		
	GS	RM		T	Wk	T × wk^3^
Intake (kg/d)						
DM^4^	10.7	10.0	0.431	0.32	<0.01	<0.01
Roughage^4^	9.54	9.08	0.433	0.47	<0.01	<0.01
Betfor^4^	1.06	1.04	0.028	0.67	<0.01	
OM	9.58	9.11	0.355	0.35	<0.01	<0.01
aNDFom	5.14	4.92	0.193	0.44	<0.01	<0.01
CP^2^	1.85^a^	1.34^b^	0.059	<0.01	<0.01	0.01
Starch^5^	0.03^b^	0.68^a^	0.027	<0.01	<0.01	<0.01
Digestibility (%)						
DM	65.0^b^	68.9^a^	1.01	<0.01	0.02	<0.01
OM	66.6^b^	70.3^a^	1.10	0.01	0.03	<0.01
aNDFom	73.1	71.8	1.27	0.45	<0.01	
CP	62.7	65.7	1.19	0.07	0.69	<0.01
Growth performance						
BW (kg)^4^, ^5^	538	540	2.61	0.54		
BW change (kg)	63.8	65.6	2.61	0.63		
ADG (g/d)	1,478	1,518	57.1	0.63		
DMI/BW^4^ (g/kg)	20.7	19.1	0.845	0.21	<0.01	<0.01
aNDFom/BW^4^ (g/kg)	9.95	9.33	0.381	0.25	<0.01	0.01
BFT^4^ (mm)	8.43	8.48	0.545	0.95		
BFT change (mm)	0.19	0.52	0.470	0.60		
Gas emissions						
Successful visit (n/heifer)	80 ± 11.2	67 ± 21.1				
CH_4_ (g/d)^4^	215	221	4.44	0.36	0.15	
CO_2_ (g/d)^4^	8,470	8,140	139.0	0.08	0.08	
CH_4_/DMI (g/kg)^4^	20.9	22.7	2.38	0.60	0.01	
CO_2_/DMI (g/kg)^4^	794	864	88.8	0.59	<0.01	
CH_4_/BW (g/kg)^4^	0.41	0.42	0.009	0.66	0.18	

a,b Within a row, means with different superscripts differ significantly (*P* < 0.05) after Tukey adjustment for multiple comparisons.

1 T = treatment; aNDFom = amylase-treated NDF expressed exclusive of residual ash; BFT = backfat thickness; Betfor = dried molassed sugar beet pulp pellets (Nordic Sugar AB, Malmö, Sweden).

2 Greatest SEM value obtained.

3 Detailed treatment × week interactions are presented in Supplemental Table S1 (see Notes).

4 Covariate included in the statistical model.

5 Back-transformed from log-transformed values (antilog scale) for interpretability.

Total DM, Betfor pellets, and roughage intake did not differ between heifers fed GS versus RM diets (Table 2). Crude protein intake was greater for GS, whereas starch intake was greater for RM. Heifers fed RM exhibited greater apparent total-tract digestibility of DM and OM, compared with heifers fed GS. Body weight, BW change, ADG, BFT, and BFT change did not differ between treatments. Detailed treatment × week interactions are presented in Supplemental Table S1 (see Notes). Post-hoc comparisons indicate that week-specific changes in roughage, aNDFom, and starch intake were inconsistent and not uniformly significant. Daily enteric CH_4_ and CO_2_ production, CH_4_ and CO_2_ yield per kilogram DMI, and CH_4_ production per kg BW did not differ between treatments (Table 2).

The total DMI for both GS and RM was consistent with expected intake for pregnant dairy heifers based on BW and dietary aNDFom concentrations (Hoffman et al., 2008). Contrary to our initial expectations, partial replacement of GS with whole-crop wheat silage did not increase aNDFom intake, despite the greater aNDFom concentration in the RM diet. Instead, RM shifted nutrient intake toward greater starch and lower CP intake, consistent with the chemical composition of whole-crop wheat silage. The similar DMI observed between treatments agrees with previous studies reporting comparable feed intake when whole-crop cereals are included in dairy cattle diets (Wallsten et al., 2009, 2010; Rustas et al., 2009, 2010, 2011).

Importantly, these intake and digestibility measurements provide representative parameter values for pregnant replacement heifers fed mixed GS-whole-crop wheat rations, a category for which empirical data remain scarce in emission inventories and LCA databases. The observed treatment × week interaction for roughage can be attributed to lower DMI in RM during wk 2 and was considered of limited biological relevance. The treatment × week interactions in digestibility parameters may partly reflect both the more intensive fecal sampling protocol during wk 4 (twice daily vs. once daily in wk 1) and differences in AIA content observed during wk 4. Such variation is plausible given the inherent heterogeneity of whole-crop cereal silages (starch-rich kernels, fiber-rich straw). Additionally, as crop maturity advances, the physical connection between these 2 fractions weakens, making kernels more prone to detachment during harvesting, mixing, or at feed-out. This can result in disproportionate kernel loss or uneven settling within the RM, lowering starch content and producing heterogeneous feed samples, which in turn may have contributed to the variation in AIA and digestibility estimates. Such variability is highly relevant for whole-farm modeling approaches, where assumptions of homogeneous feed composition may underestimate uncertainty in nutrient flows and associated GHG emissions.

Contrary to our hypothesis, partial replacement of GS with whole-crop wheat silage did not affect enteric CH_4_ emissions. This was likely because DMI, the primary driver of enteric CH_4_ production, was similar between treatments (Mills et al., 2003; Ramin and Huhtanen, 2013; Beauchemin et al., 2022). These findings support the use of DMI-based emission factors for replacement heifers in national inventories and LCA studies. Enteric CH_4_ per kilogram DMI was similar across both treatments despite the differences in chemical composition, including OMD, CP, and starch intake (Nielsen et al., 2013; Niu et al., 2021). Although RM increased starch intake, the starch concentration of the whole-crop wheat silage was lower than expected (Rustas et al., 2011), which may have limited effects on rumen fermentation and CH_4_ yield (Van Kessel, 1996; Hook et al., 2011). The higher CP intake in the GS group could also have contributed to a lower enteric CH_4_ production (Mills et al., 2003; Yan et al., 2006; Nielsen et al., 2013). Together, these opposing dietary characteristics may explain the absence of treatment effects on enteric CH_4_ emissions. Potential effects of BW and breed on enteric CH_4_ production were controlled through the blocking design, minimizing confounding in the treatment comparison (Hoffman et al., 2008; Yan et al., 2006).

We did not observe any difference in BW, BW change, ADG, or absolute BFT between animals in GS and RM. The observed ADG exceeded the ∼0.75 kg/d guideline for heifers targeting 24 mo of age at calving (Wathes et al., 2014) and the >1.1 kg/d threshold linked to excess fat accumulation (Akins, 2018). However, given the short duration of the experiment, the mid-gestation stage of the heifers, and the limited increase in BFT, we do not expect adverse effects on subsequent performance. An increase in BCS at calving from 4 to 6 (9-point scale) has been shown not to affect dystocia scores, while positively influencing calf birth weight and increasing the proportion of heifers exhibiting estrus and becoming pregnant by 40 and 60 d of the subsequent breeding season (Larson, 2007). Additionally, a 1-mm change in BFT is associated with a ∼5-kg change in total body fat (Schröder and Staufenbiel, 2006). The changes observed in the current experiment correspond to 0.95 and 2.60 kg total body fat gain (±2.35 kg), suggesting that both treatments showed similar growth rates without excessive total body fat accumulation. Thus, the resulting BW increase can be attributed to the heifers' development (e.g., skeletal and muscle development).

From a systems perspective, improved growth efficiency shortens the nonproductive rearing phase, a major contributor to lifetime GHG emission intensity (Clasen et al., 2024). The lack of treatment effect on DMI and ADG suggests that whole-crop wheat can, when required, replace 50% of GS (DM basis) in dairy heifer diets without increasing feed required to support growth or affecting animal performance or enteric CH_4_ emissions. Future studies should evaluate higher inclusion rates of whole-crop cereal silage, including complete replacement of GS, to support feeding strategies during drought-induced forage shortages. The high growth rates observed in this study further highlight the importance of ad libitum feeding strategies for replacement dairy heifers during late gestation. In the present experiment, all animals were at the start of the second trimester of pregnancy (3.9 ± 0.95 mo of gestation), a period during which animals must support both maternal growth and fetal development (Larson, 2007). Achieving adequate growth before calving, without an excessive increase in total body fat reserves, is particularly important because nutrient requirements increase substantially during early lactation, when primiparous cows must simultaneously support lactation, continued growth, and reproductive recovery (Larson, 2007). The animals in the present experiment produced 212 to 221 g enteric CH_4_/d, corresponding to about half of the expected emissions from a high-producing Swedish Holstein dairy cow fed a diet based on GS (Managos et al., 2025). Although individual heifers emit less CH_4_ than lactating cows, replacement heifers contribute substantially to whole-farm GHG emissions because of the length of the rearing period and herd replacement requirements (Clasen et al., 2024; Sommerseth et al., 2024). These findings emphasize the value of optimized nutritional strategies to improve growth efficiency while limiting GHG emissions (Sommerseth et al., 2024). This study also provides empirical inputs for LCA of dairy systems, including DMI, digestibility, enteric CH_4_ emissions, and growth performance during the rearing phase, although additional studies are needed to strengthen system-level estimates.

In conclusion, whole-crop wheat silage can partially replace GS in dairy heifer diets without affecting growth performance or enteric CH_4_ emissions. These findings support flexible forage strategies when GS availability is limited and provide empirical data for environmental assessments of dairy systems.
